# Gilthead Seabream Liver Integrative Proteomics and Metabolomics Analysis Reveals Regulation by Different Prosurvival Pathways in the Metabolic Adaptation to Stress

**DOI:** 10.3390/ijms232315395

**Published:** 2022-12-06

**Authors:** Cláudia Raposo de Magalhães, Ana Paula Farinha, Gavin Blackburn, Phillip D. Whitfield, Raquel Carrilho, Denise Schrama, Marco Cerqueira, Pedro M. Rodrigues

**Affiliations:** 1 Centre of Marine Sciences (CCMAR), Universidade do Algarve, Campus de Gambelas, 8005-139 Faro, Portugal; 2 Universidade do Algarve, Campus de Gambelas, 8005-139 Faro, Portugal; 3 Glasgow Polyomics, University of Glasgow, Glasgow G61 1BD, UK

**Keywords:** aquaculture, bioinformatics, fish welfare, hypoxia, mass spectrometry, multiomics, net handling, overcrowding

## Abstract

The study of the molecular mechanisms of stress appraisal on farmed fish is paramount to ensuring a sustainable aquaculture. Stress exposure can either culminate in the organism’s adaptation or aggravate into a metabolic shutdown, characterized by irreversible cellular damage and deleterious effects on fish performance, welfare, and survival. Multiomics can improve our understanding of the complex stressed phenotype in fish and the molecular mediators that regulate the underlying processes of the molecular stress response. We profiled the stress proteome and metabolome of *Sparus aurata* responding to different challenges common to aquaculture production, characterizing the disturbed pathways in the fish liver, i.e., the central organ in mounting the stress response. Label-free shotgun proteomics and untargeted metabolomics analyses identified 1738 proteins and 120 metabolites, separately. Mass spectrometry data have been made fully accessible via ProteomeXchange, with the identifier PXD036392, and via MetaboLights, with the identifier MTBLS5940. Integrative multivariate statistical analysis, performed with data integration analysis for biomarker discovery using latent components (DIABLO), depicted the 10 most-relevant features. Functional analysis of these selected features revealed an intricate network of regulatory components, modulating different signaling pathways related to cellular stress, e.g., the mTORC1 pathway, the unfolded protein response, endocytosis, and autophagy to different extents according to the stress nature. These results shed light on the dynamics and extent of this species’ metabolic reprogramming under chronic stress, supporting future studies on stress markers’ discovery and fish welfare research.

## 1. Introduction

Over the past 20 years, global aquaculture has been thriving and developing toward the critical goals of environmental sustainability, economic sustainability, and societal sustainability. Producing more food (i.e., calories, proteins, amino acids) per unit of land area is the current model for overcoming global population expansion. With the continuous intensification of aquaculture, the welfare of farmed fish is becoming a key issue, for which stress management has become a powerful tool to continue improving the sector’s sustainable growth [1]. Exposure to minor stressors is known to promote fish resilience, while major stressors can affect whole-animal performance and, in more severe cases, survival [2]. Fish respond to stressful stimuli through an elaborate endocrine machinery that provides the chemical mediation of a hypothalamic combined signal through the action of glucocorticoids, to manage the production and expenditure of energy and allow for a proper fight-or-flight response [3]. The liver is the central organ in the energy management adaptation response, responsible for processes such as the synthesis of glucose and fatty acids degradation, to compensate for coping mechanisms. However, if the severity of the stimulus increases, the stress response can shift to an energy conservation state, and high-energy costly biological functions, such as immunity and growth, are suppressed, which might compromise fish welfare [4]. At the hepatocyte level, cells undergo immediate changes to help their metabolism adapt and to protect themselves against potential damage. This process is orchestrated through a multilayered cellular program, which involves the concerted action of diverse stress-signaling pathways regulated at different levels of biological organization [5]. The cellular stress response was recently reviewed in fish subjected to salinity [6] and temperature stress [7]. However, studies addressing the specific signaling pathways supporting the cellular stress response at different organizational levels are scarce.

High-throughput technological advances, such as “omics” approaches, allow for a deeper understanding of the intricate network of signaling pathways involved in the regulatory mechanisms responsible for the specific metabolic adaptation to different stressors. Proteins and metabolites represent the downstream outcome of an organism’s genome and its interaction with the environment. Thus, untargeted proteomics and metabolomics analyses can provide a high-resolution snapshot of a fish subjected to a certain stressful stimulus. Proteomics has been extensively used to study fish hepatic metabolism in response to different stressors [8,9,10,11,12,13,14,15]. On the other hand, the use of untargeted metabolomics in fish stress research, although less explored, has been occurring in recent years [16,17,18]. However, integrated multiomics studies are still limited. Combining omics strategies permits a more holistic understanding of the interrelationships of the active biomolecules and their corresponding functions, and it is becoming a powerful tool for life science research. However, in fish and aquaculture research, this approach is still hampered by the lack of genomic data, analytical tools, and comprehensive databases, among other aspects [19]. Notably, few studies with different fish species, combining different omics platforms, have provided remarkable insights into nutrition [20] and exposure to environmental stressors [21,22,23,24] and pathogens [25,26,27].

Gilthead seabream (*Sparus aurata*) is one of the most produced finfish species in Mediterranean aquaculture and the fourth major species produced in Europe [28]. Therefore, molecular insights into the stress physiology of this species could provide the industry with deeper knowledge on welfare conditions, eventually devising stress-mitigation strategies by conventional and modern farming to develop improved recommendations on best practices. To the best of our knowledge, the present work is the first multiomics study to portray signaling and metabolic pathway perturbations in challenged gilthead seabream. Herein, we subjected gilthead seabream adults to different challenges with different severities, simulating standard practices and conditions in an aquaculture farm, such as overcrowding, net handling and hypoxia, and conducted an integrated proteomics and metabolomics analysis of the liver tissue through high-resolution liquid chromatography coupled with mass spectrometry (LC-MS/MS). These data will link the interplay of different stress-signaling pathways to complex stress variation and enhance our understanding of biological stress pathways decoded by the animal stress response. We henceforward envisage an increase in the use of more-integrated approaches within fish stress research built upon the knowledge attained from comprehensive and system-wide studies to improve aquaculture sustainability and bring about a less-damaging food system.

## 2. Results

### 2.1. Proteomics and Metabolomics Data Overview

Proteomics analysis of gilthead seabream liver led to a mean of 40 ± 2% of peptide spectrum matches (PSM) out of a mean of 27,122 ± 917 total spectra per MS/MS sample (Table S1). About 1738 proteins were identified with a probability higher than 99% to achieve a false discovery rate (FDR) of <1% assigned by the protein prophet algorithm, with at least three peptides, on the Scaffold software v.4.11.1 (Proteome Software Inc. Portland, OR, USA) (Table S2). From these, 1443, 1435, and 1418 reproducible proteins, present in at least four out of six replicates were selected from OC, NET, and HYP trials, respectively, for further statistical analyses. On the other hand, metabolomics analysis detected 3393, 5846, and 3616 peaks for the OC, NET, and HYP liver samples, respectively, from which 840, 1189, and 894 metabolite-like signals were annotated on the basis of mass and retention time, and 84, 112, and 89 were identified against authentic in-house standards (Table S4).

A differential analysis of the liver proteomes between the control and challenged groups revealed 40, 349, and 46 differentially abundant proteins (DAPs) (Student’s *t*-test, *p* < 0.05, 5% FDR) among the OC, NET, and HYP trials, respectively, from which 20, 202, and 20 were upregulated in the challenged group, and 20, 147, and 26 were downregulated (Table S3). A Student’s *t*-test with 5% FDR correction of the metabolomic data identified 40 and 58 peaks corresponding to confidently identified metabolites, significantly changing the abundance (*p* < 0.05) in NET- and HYP-challenged groups, compared with the corresponding control group. No differentially abundant metabolites (DAMs) were identified between control fish and challenged fish in the OC trial (Table S5).

**Figure 1:**
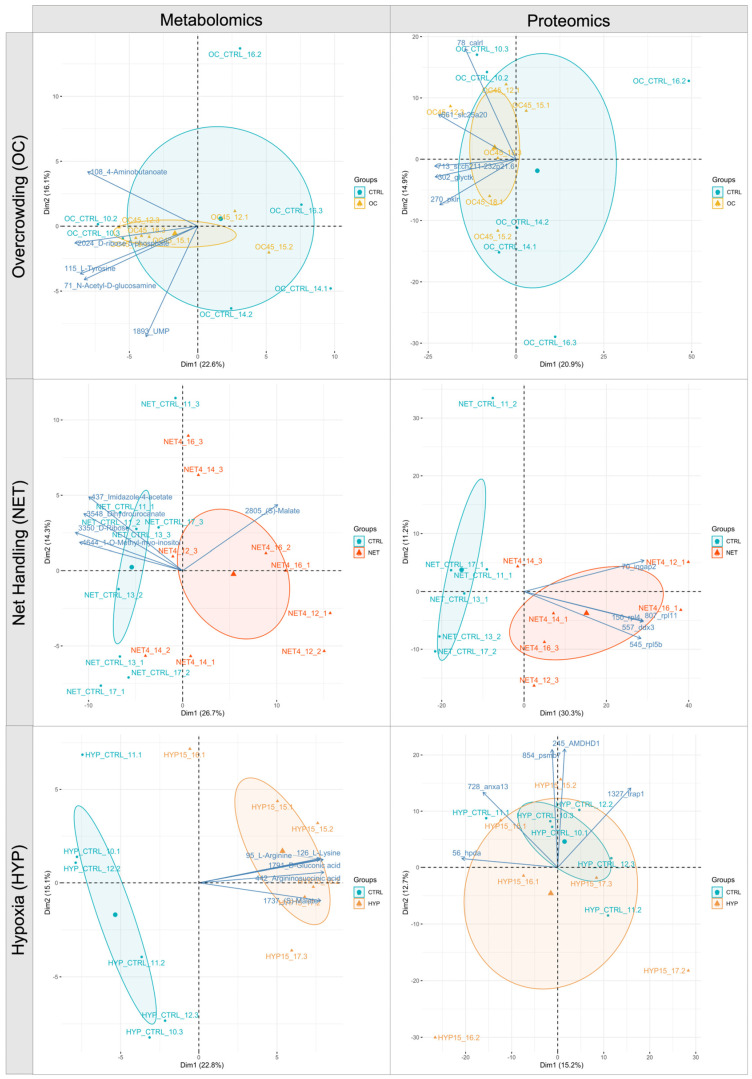
Principal component analysis (PCA) biplots of the liver proteomics and metabolomics data of gilthead seabream subjected to overcrowding (OC), net-handling (NET) and hypoxia (HYP) challenges. Each point represents a biological replicate’s projection, and experimental groups within trials are represented by a unique color, as indicated in each legend. The largest point represents the group mean. The axes’ percentages indicate the proportions of explained variance. The arrows depict the top-five most weighted variables.

The score scatter plots (Figure 1) of the principal component analysis (PCA) conducted with the reproducible set of proteins from each trial showed that the first two principal components were able to capture 35.8%, 41.5%, and 27.9% of the total variability of OC, NET, and HYP trials data, respectively. A clear separation between groups was obtained for the NET data along the PC1 axis. The plots display the top-five features with the highest loading values as variable arrows. The PCA of the metabolomics data, conducted with the identified and single-annotated metabolites, showed that PC1 and PC2 were able to explain 38.7%, 41%, and 37.9% of the variability within OC, NET, and HYP datasets, respectively, achieving a better group separation for the NET and HYP trials than the PCA did, according to the proteomics data. In Figure 1, the top-five features with the highest loading values are displayed as variable arrows in the biplots.

### 2.2. Integrated Stress Response Analysis

**Figure 2:**
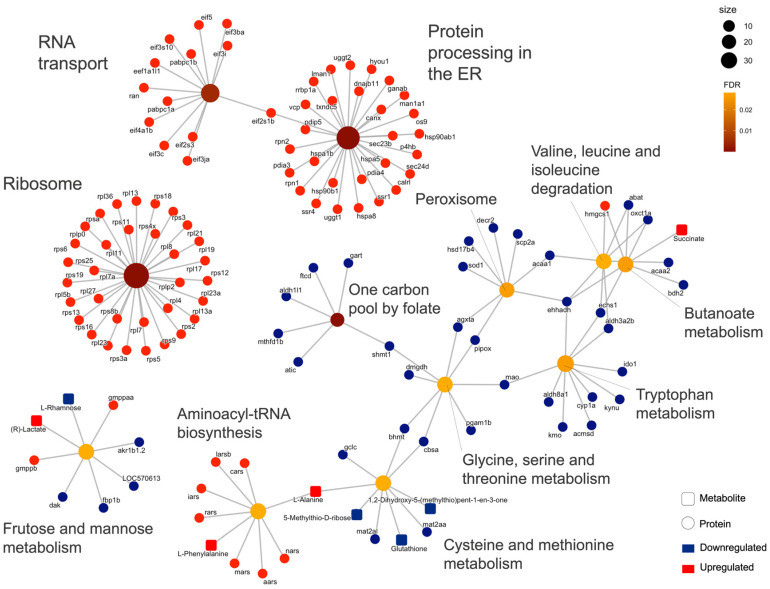
A gene-concept network of enriched KEGG terms (FDR < 0.05) within the differential abundant proteins (DAPs) and metabolites (DAMs) identified in the liver of gilthead seabream subjected to the net-handling (NET) challenge. Central nodes represent the enriched term, with color and size representing FDR and the number of associated biomolecules, respectively. The concept nodes represent biological concepts, where shape corresponds to the omics modality and color to the regulation of that biomolecule, determined by Student’s *t*-test with FDR controlled at 0.05.

An overrepresentation analysis (ORA) of differential proteins and metabolites from NET fish liver resulted in 12 overrepresented KEGG terms with FDR < 0.05, the three most enriched being ribosome (FDR = 1.032 × 10^−22^), protein processing in endoplasmic reticulum (ER) (FDR = 2.384 × 10^−14^) and one-carbon pool by folate (FDR = 0.002) (Figure 2; Table S6). Regarding the HYP analysis, 11 enriched terms were revealed: ABC transporters (FDR = 4.926 × 10^−8^), aminoacyl-tRNA biosynthesis (FDR = 0.003) and alanine, aspartate, and glutamate metabolism (FDR = 0.003) were the three most enriched (Figure S1). No joint pathway analysis (JPA) was performed for the OC data because no DAMs were identified by univariate hypothesis testing.

**Figure 3:**
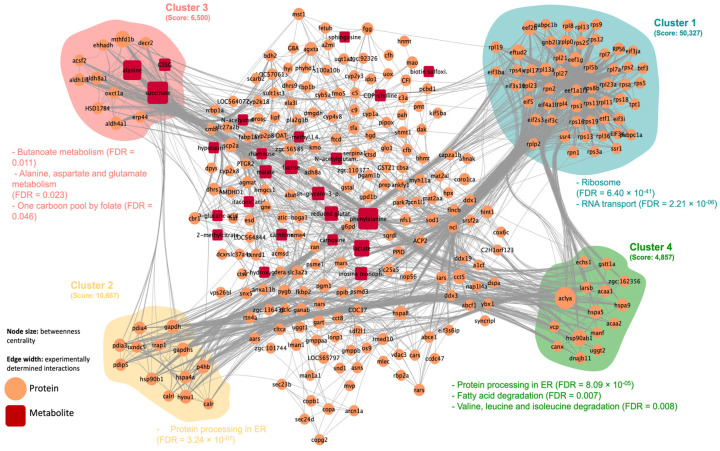
Metabolic reaction network generated with the differential abundant proteins and metabolites identified in the liver of gilthead seabream subjected to a net-handling challenge. Node shape and color represent the type of biomolecule, according to the legend. Edges represent functional linkages between them. The highlighted clusters, depicted with the MCODE plugin within Cytoscape software, represent the most interconnected regions, with the corresponding overrepresented in KEGG terms (FDR < 0.05).

A metabolic reaction network (metabolite protein) was generated for each trial, i.e., NET and HYP, with the corresponding DAPs and DAMs. The topological analysis on Cytoscape revealed a network with 296 nodes, 3176 edges, and a clustering coefficient of 0.428, for the NET trial, with four main clusters highlighted by MCODE plugin (Figure 3). The top three nodes with the highest betweenness centrality were succinate, phenylalanine, and alanine. The JPA conducted on each cluster individually showed that the enriched KEGG terms were involved mainly in translation, folding processes, and amino acid metabolism (Figure 3). The HYP trial DAPs and DAMs generated a network with 71 nodes, 438 edges, a clustering coefficient of 0.704, and 1 highly interconnected cluster identified by MCODE. Proteins in this cluster were enriched mainly in KEGG terms related to amino acids, nucleotides, and lipid metabolism. The highest betweenness centrality values were attributed to phosphate, ATP, and ADP metabolites (Figure S2).

**Figure 4:**
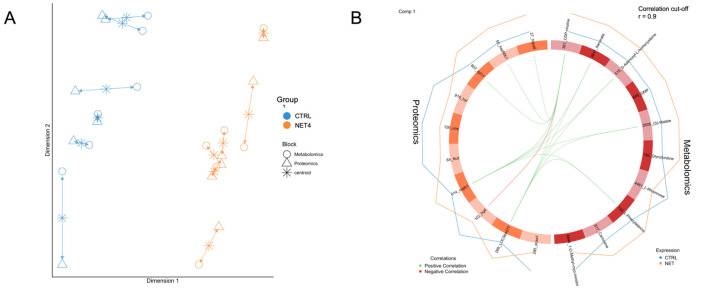

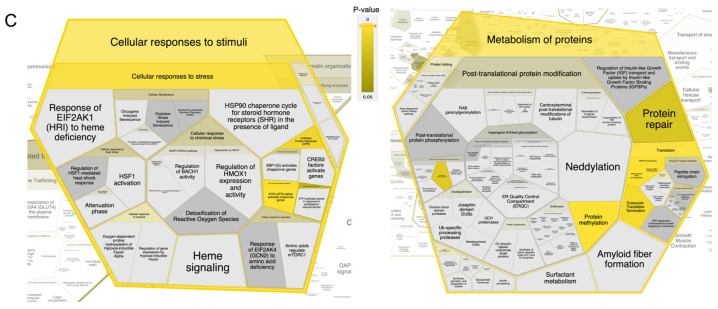
Integrated proteomics and metabolomics analysis, conducted with DIABLO, of the liver of gilthead seabream subjected to the net-handling challenge (NET). (**A**) Arrow plot of the separation between groups achieved with the first two components of the DIABLO model. Different shapes represent different data modalities. (**B**) Circos plots representing the Pearson correlation (correlation cutoff = 0.9) between the 10 most-discriminatory proteins and metabolites selected by the first component of the DIABLO model. (**C**) Voronoi plots obtained with a REACTOME analysis tool represent two of the most overrepresented high category terms (FDR < 0.05), among the upregulated features selected by DIABLO. The *p*-value scale indicated in the figure legend corresponds to the adjusted *p*-value.

DIABLO was used to perform a correlation analysis between both data modalities and to depict each trial’s 20 most-discriminatory features (10 metabolites and 10 proteins). The arrow plot (Figure 4A) of the first two components of the NET DIABLO model showed that group separation was achieved over the first dimension and that replicates’ variability was consistent across omics datasets. Proteomics and metabolomics data showed a Pearson correlation of 0.95 on the first component, with six proteins and five metabolites showing r > 0.9, as demonstrated in the circos plot from Figure 4B. The features from the first component were then subjected to an ORA on the REACTOME database (Table S7), according to their expression patterns, to depict the biological functions and pathways that were most affected by the NET challenge (Figure 4C). A total of 106 terms presented FDR < 0.05, for the upregulated proteins and metabolites, where transport of small molecules (FDR = 2.19 × 10^−2^), cellular responses to stimuli (FDR = 2.19 × 10^−2^) and metabolism of proteins (FDR = 2.19 × 10^−2^) were the three most enriched higher-level terms. In the case of the downregulated features, 10 lower-level terms showed FDR < 0.05, being mostly implicated in the metabolism of certain amino acids and peroxisomal processes. Regarding the HYP trial, the final DIABLO model calculated two components that could separate control from the hypoxia challenged group along the dimension 1 axis, according to the arrow plot in Figure S3A. The Pearson correlation between both data modalities on the first component was 0.96, while four proteins and six metabolites among the selected features demonstrated r > 0.8 (Figure S3B). A REACTOME overrepresentation analysis of the selected upregulated and downregulated features (Figure S3C) showed that 84 terms were upregulated (FDR < 0.05), the transport of small molecules (FDR = 6.91 × 10^−3^), metabolism (FDR = 2.16 × 10^−2^), and cellular responses to stimuli (FDR = 6.47 × 10^−2^) being the most enriched. On the other hand, among the 77 downregulated terms with FDR < 0.05, the three most enriched were mainly integrating energy metabolism, protein repair, and the metabolism of vitamins and cofactors.

## 3. Discussion

The stress response is a regulatory mechanism conserved among vertebrates, which comprises a cascade of events from the molecular to the whole individual level. This internal disturbance can either culminate in the organism’s adaptation, characterized by a compensating stress response (i.e., reallocation of energy resources with reversible physiological damage and potentially improved fish fitness), or scale up to a metabolic shutdown, characterized by irreversible cellular damage and resulting in permanent deleterious effects on fish performance and survival. What defines this threshold depends on individual- (e.g., species, age, previous experiences, coping styles) and/or stressor-related factors (e.g., severity), increasing the difficulty of defining a stressed phenotype in fish [29]. The molecular regulation of this complex response occurs at different biological levels, and therefore, the integrated approach employed in this study provides a systemic perspective of the main interactions between the hepatic proteome and metabolome and unveils the main pathways underpinning this species’ stress response to different challenges. In aquatic animals, the liver is responsible for the storage, production, and reallocation of energy resources and is one of the most profoundly affected organs during a stress response [30]. It has been shown that the magnitude of the liver response greatly depends on the severity of the stimulus [14], specifically acute vs. chronic stimuli. At the cellular level, this translates into stress responses that can be manifold, ranging from the activation of survival pathways, which are geared toward helping the cell recover from the insult, to eliciting programmed cell death. The cell’s fate critically relies on its ability to mount an appropriate stress response.

In this study, the numbers of dysregulated proteins and metabolites in the liver suggested that net handling was the challenge that induced the most intense metabolic reprogramming in gilthead seabream (349 DAPs and 40 DAMs vs. 46 DAPs and 58 DAMs in the HYP trial and 40 DAPs and 0 DAMs in the OC trial), most likely related to its severity (1^1/2^ months compared with the 48 h of the hypoxia challenge). These results might also suggest a multistress effect, as recently proposed [31], which might have resulted from a synergetic combination of net handling and air exposure. Previous plasma and liver analyses conducted on these same fish, published elsewhere [14,32], also verified this same difference in response to these different challenges. According to the ORA performed with REACTOME on the NET proteins and metabolites selected by the DIABLO model (Table S7), terms related to the metabolism of amino acids, amino acid transport across the plasma membrane, unfolded protein response (UPR), and translation were included among the most enriched ones. Notably, pathways related to the catabolism of amino acids were mainly downregulated, whereas pathways related to the biosynthesis of a specific set of amino acids were essentially upregulated. Simultaneously, amino acid transport, translation, and UPR were upregulated (Figure 4C). These results suggest that net handling most likely induced cellular stress and that challenged fish coped with the stress by activating this prosurvival pathway, i.e., UPR. UPR is directly and indirectly related to the other overrepresented pathways through different regulatory processes, in an intricate network of signaling pathways to ensure cell survival and tissue homeostasis, as will be further discussed. The protein–metabolic interaction network also demonstrated this interconnection between these pathways (Figure 3).

**Figure 5:**
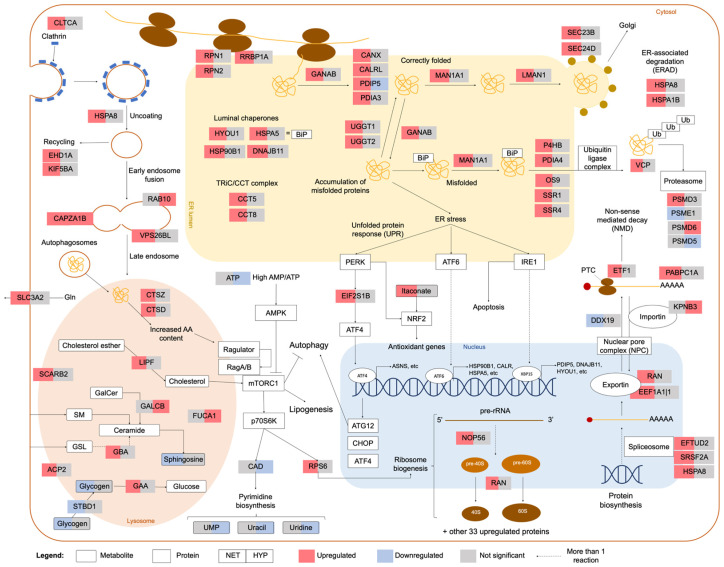
Overview of the cellular processes and signaling pathways affected by net handling and hypoxia in gilthead seabream hepatocytes. The proteins and metabolites represented were differentially different in abundance, according to a Student’s *t*-test with FDR controlled at 0.05.

During normal and stressful circumstances, secreted proteins undergo maturation in the ER before being exported to the Golgi apparatus, if properly assembled. Cellular stress may disturb this process, resulting in the accumulation of unfolded/misfolded proteins in the ER (ER stress) and culminating in the orchestration of the UPR. This response is initiated by three ER-resident molecular proteins, most notably inositol-requiring protein-1 (IRE1), protein kinase RNA (PKR)-like ER kinase (PERK), and activating transcription factor 6 (ATF6), which will activate three intracellular signal transduction pathways. PERK phosphorylates eukaryotic initiation factor 2 (eIF2α) allows the translation of activating transcription factor 4 (ATF4), which may, in turn, activate the transcription of chaperones and other proteins involved in the regulation of apoptosis (e.g., C/EBP Homologous Protein (CHOP)), autophagy (e.g., autophagy related 12 (ATG12)), and amino acid metabolism (e.g., asparagine synthetase (ASNS)). PERK can also directly phosphorylate the transcription factor NF-E2-related (NRF2), which induces the expression of antioxidant genes [33]. In this study, proteins eIF2α and ASNS were upregulated in NET-challenged fish. Accordingly, in largemouth bass exposed to heat stress, eIF2α gene was also reported to be upregulated, along with other UPR-related genes [34]. In contrast, ATF6 is first activated in the Golgi and then acts as a transcription factor of chaperones, such as the 78 kDa glucose-regulated protein (BiP/GRP78/HSPA5) and the 94 kDa glucose-regulated protein (HSP90B1), x-box-binding protein 1 (XBP1), and genes involved in the ER quality control machinery (e.g., calreticulin (CALRL)) [33]. HSPA5 and HSP90B1 were upregulated in NET-challenged fish, together with five other heat-shock proteins (HSPs) (Table S3). In fact, the upregulation of proteins from the HSP family is a commonly reported response to stress in fish [30,35]. Corroborating these results, these proteins were previously reported to be upregulated in the liver of these same fish, after a gel-based proteomics analysis, along with a positively correlated expression pattern of the corresponding genes, assessed by real-time polymerase chain reaction (RT-PCR) [14]. Lastly, IRE1 catalyzed the alternative splicing of XBP1 mRNA, leading to the expression of the XBP1 transcription factor. Subsequently, this transcription factor activated the expression of numerous genes, encoding proteins from the ER quality control machinery (e.g., hypoxia upregulated 1 (HYOU1), DnaJ heat-shock protein family member B11 (DNAJB11), protein disulfide-isomerase 6 (PDIP5)), ER-associated degradation (ERAD), and lipid synthesis [33]. In summary, in NET-challenged fish, several proteins that participated in these processes, i.e., UPR, protein folding, quality control, protein tracking to Golgi (COPII-dependent anterograde transport), and ERAD, were upregulated, suggesting that NET-challenged fish’s hepatic cells activated UPR to attempt to improve the balance between protein load and folding capacity, by translating specific proteins, and consequently to attenuate ER stress. The upregulation of different aminoacyl-tRNA synthetases and several translation-related proteins (Table S3) corroborated this hypothesis. Furthermore, previous studies have reported an upregulation of ER stress in seawater-transferred rainbow trout livers [36], common carp exposed to hydrogen peroxide [37], rainbow trout following overcrowding stress [38], and gilthead seabream exposed to low temperatures [39]. On the other hand, PDIP5 was found to be downregulated in hypoxia-exposed fish, exposing again the divergent responses of NET and HYP fish according to the challenge severity. A schematic diagram summarizing these stress mechanisms and the associated dysregulated proteins is depicted in Figure 5.

The ERAD system mediates the removal of incorrectly folded proteins in a multistep process involving the recognition and targeting of substrates, followed by ubiquitination, retrotranslocation, and degradation [40]. After ubiquitination, protein recycling can occur through autophagy and/or the ubiquitin-proteasome system (UPS) [41]. NET fish presented 12 proteins involved in the ERAD process that were found to be upregulated, suggesting that protein degradation was stimulated in challenged fish. Nevertheless, two proteins belonging to the proteasome showed ambiguous expression patterns (PSMD3 was upregulated, whereas PSME1 was downregulated). The same was verified for HYP fish (PSMD6 was upregulated, whereas PSMD5 was downregulated) (Figure 5). In OC fish, proteasome subunit beta type-6 (PSMB6) was upregulated. Changes in proteasome subunits were reported in Atlantic cod in response to *V. anguillarum* infection [42] and high-temperature exposure [43]. Interestingly, heat-shock protein family A (Hsp70) member 8 (HSPA8), an upregulated protein in NET-challenged fish’s ERAD response, is involved in a process called “chaperone-mediated autophagy”, which entails the direct delivery of cytosolic proteins, targeted for degradation, to the lysosomes. However, this cellular function has been poorly described in fish because, until recently, it was presumed to be exclusive to mammals and birds [44,45]. Moreover, the upregulation of HSPA8 was also observed in Senegalese sole following repeated handling stress [46] and in gilthead seabream fed maslinic-supplemented diets [47].

**Figure 6:**
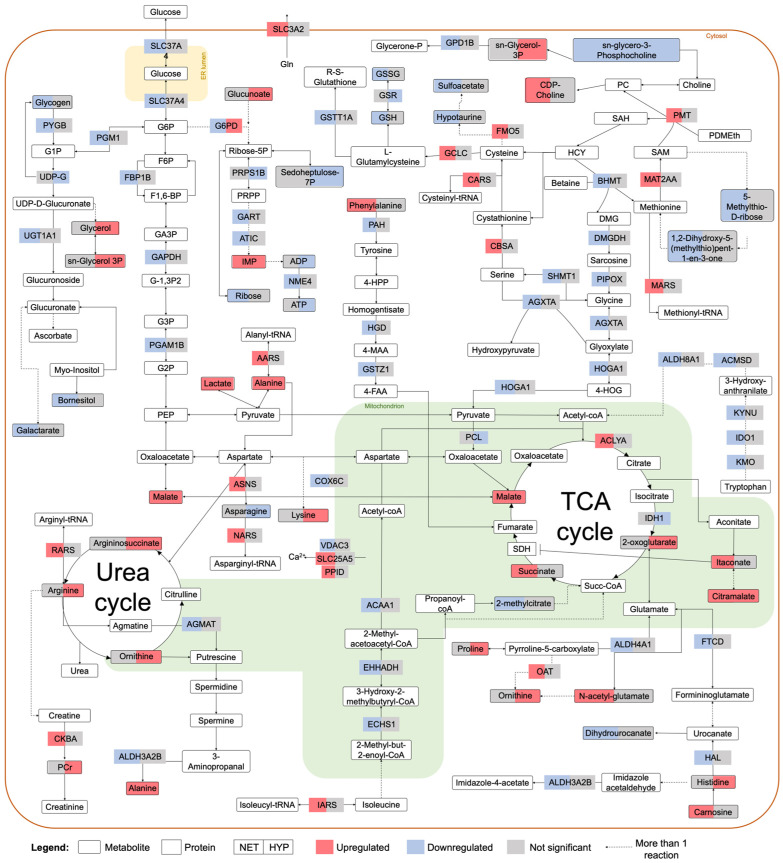
Overview of the metabolic pathways affected by net handling and hypoxia in gilthead seabream hepatocytes. The proteins and metabolites represented were significantly different in abundance, according to a Student’s *t*-test with FDR controlled at 0.05.

Macroautophagy, the best-described form of autophagy, is a key pathway in stress-induced metabolic adaptation and damage control. It is responsible for degrading intracellular substrates, such as organelles or proteins, in bulk or selectively by encasing them in double-membraned vesicles, called autophagosomes, which are then delivered to the lysosome [48]. On the other hand, the degradation and recycling of extracellular substrates are facilitated by endocytosis. The dynamic remodeling of the plasma membrane proteome is crucial to the cellular adaptation to stress, as many signaling cascades originate at the cell surface receptors. In clathrin-mediated endocytosis (CME), responsible for most of this flux, formed vesicles later fused with lysosomes for content degradation. CME also contributes to the uptake of material such as metabolites, hormones, and other proteins from the extracellular space [49]. The degradation in the lysosome is then mediated by the action of hydrolases, including cathepsins. Both NET- and HYP-challenged fish had upregulated proteins involved in these pathways (Figure 6), suggesting that endocytosis also participated in the cellular stress response to these challenges. Cathepsin d (CTSD) has previously been reported to be upregulated in the liver of trout following an acute stressor [50]. Different cathepsins were also upregulated in fasting gilthead seabream [51]. Additionally, glycogen is also delivered to the lysosomes by selective autophagy, in a mechanism known as “glycophagy”. Glycogen is the main carbohydrate store in fish liver, and a rapid generation of glucose after acute stimuli is usually achieved by glycogen breakdown [52]. The lysosomal hydrolytic degradation of glycogen in the liver has been proposed as an alternative route, occurring in parallel to the canonical glycogenolysis pathway in cytosol, to meet high-circulating-glucose demands for systemic utilization. Contrary to glycogenolysis, glycophagy produces nonphosphorylated glucose that can be more rapidly used [53]. However, in fish, these mechanisms are still poorly described. Nonetheless, glycogen phosphorylase B (PYGB), responsible for catalyzing the phosphorolysis of glycogen in the first step of glycogenolysis, was found to be downregulated in NET fish (Figure 6). Alongside, lysosomal alpha-glucosidase (GAA), responsible for the breakdown of glycogen in the lysosomes, was upregulated (Figure 5), suggesting that glycogen degradation was occurring mainly at the lysosomal level. Concomitantly, glycogen levels in NET fish were significantly downregulated, as previously assessed by commercial kits and published in another work [14], whereas plasma glucose levels were significantly upregulated [32]. Lysosomes are the control center of catabolic and anabolic processes in eukaryotic cells, recycling the building blocks of the cargo delivered by endocytic and autophagic pathways and regulating cell physiology. In fact, lysosomes have been recently investigated as signaling hubs and central organelles in the mammalian cellular stress response [54,55,56]. In fish, lysosomal stability has been used as a bioindicator of environmental toxicity [57].

One of the most important regulators of cell metabolism, growth, and proliferation is the mechanistic target of rapamycin complex 1 (mTORC1), which is activated mainly by growth factors and both high amino acid cytosolic and intraluminal content. When activated, mTORC1 is recruited to the cytosolic face of the lysosomal membrane. Henceforth, a complex cascade of tightly regulated reactions switches the metabolism toward anabolic processes such as ribosome biogenesis, protein synthesis, glucose metabolism, and nucleotide and lipid synthesis and inhibits catabolic processes such as autophagy. At a downstream level, mTORC1 activates p70 ribosomal protein S6 kinase1 (p70S6K), which further phosphorylates ribosomal protein S6 (RPS6) and carbamoyl phosphate synthetase 2-aspartate transcarbamoylase-dihydroorotase (CAD), responsible for inducing ribosome biogenesis and de novo pyrimidine biosynthesis, respectively [58]. Results for NET fish are contradictory regarding mTORC1 signaling, in that RPS6 and several other proteins involved in ribosome biogenesis (35 proteins in total) were upregulated (Figure 5), whereas some proteins participating in purine and pyrimidine biosynthesis were downregulated. On the one hand, the production of one-carbon units required for de novo purine biosynthesis was downregulated (Figure 2), and on the other hand, the glutamine-leucine SLC3A2-SLC7A5 antitransporter and leucyl-tRNA synthetase (LARS), two proteins known to activate mTORC1 [59,60], were found to be upregulated in these fish. Hence, more analyses would be needed to infer this pathway’s regulation on NET fish. Undeniably, mTOR-signaling regulation in fish has been extensively described in muscle growth [61,62] and nutrition studies [63,64,65]. In stress-related studies, genes involved in mTOR signaling were found to be upregulated in the liver of gilthead seabream subjected to hyper- and hypo-osmotic challenges [66]. However, the mTORC1 regulatory mechanisms in fish during stress remain largely elusive. In contrast, in HYP fish, results suggest that this pathway was potentially downregulated, as CAD and pyrimidine biosynthesis-related proteins were downregulated, as were adenosine triphosphate (ATP) levels (Figure 5). Additionally, asparagine, a nonessential amino acid known to stimulate mTORC1 signaling [67], was similarly downregulated (Figure 6). Low ATP levels inhibit mTORC1 because this complex responds to cellular energy status via the heterotrimeric adenosine monophosphate (AMP)-activated protein kinase (AMPK). When the cellular ATP:AMP ratio is low, which is common in hypoxia stress because of a shortage in oxygen and consequently an impaired mitochondrial respiratory chain, AMPK inhibits mTORC1 to conserve energy because protein biosynthesis consumes the lion’s share of energy and cellular resources [68]. In the liver of goldfish subjected to hypoxia, the authors reported that AMPK activity increased by ~5.5-fold, with no increase in protein abundance, suggesting that changes in AMPK activity are most likely due to post-translational modifications (PTM). These were accompanied by an increase in the ATP:AMP ratio and a decrease in protein synthesis [69]. The same was verified for rainbow trout [70]. The inhibition of the mTOR-signaling pathway was reported in Artic char exposed to hypoxia, precisely 15% of air saturation, along with a decrease in the rate of protein synthesis [71]. In humans, the dysregulation of the mTOR-signaling pathway has been associated with aging and several diseases [72].

During aerobic conditions, pyruvate, resulting from glycolysis, is introduced into the tricarboxylic acid (TCA) cycle, which theoretically generates 36 ATP molecules via a respiratory chain in the mitochondria [73]. Inadequate tissue oxygenation inhibits the process of oxidative phosphorylation (OXPHOS), and fish can generate ATP only through substrate-level phosphorylation, which includes converting pyruvate into lactate in the cytosol, and phosphate transfer from intermediates such as creatine phosphate (PCr) [74]. Accordingly, in this study, pyruvate carboxylase (PCL), which catalyzes the conversion of pyruvate into oxaloacetate to enter the TCA cycle, was downregulated in hypoxia-exposed fish, whereas lactate and creatine phosphate were upregulated (Figure 6). The downregulation of isocitrate dehydrogenase (IDH1), an enzyme of the TCA cycle, in these fish also corroborates the suppression of this pathway, together with the upregulation of glucogenic amino acids (e.g., alanine, arginine, proline), which enter the TCA cycle to further produce glucose (Figure 6). Concomitantly, a 1H-NMR-based metabolomics study revealed an upregulation of lactate, alanine, and PCr in hypoxia-exposed common carp [75]. Interestingly, in NET fish, lactate was likewise upregulated in challenged fish, and cytochrome c oxidase subunit 6C (COX6C), the terminal enzyme of the mitochondrial respiratory chain, was downregulated, suggesting that OXPHOS was also downregulated in net-handled fish (Figure 6). Another metabolite involved in OXPHOS is succinate, a substrate of the TCA cycle, which is at the crossroads of numerous other metabolic pathways, such as the degradation of branched-chain amino acids (BCAAs), GABA shunt, and post-translational modifications, among others. Succinate was found to be upregulated in NET fish (Figure 6), which might be explained by the fact that succinate dehydrogenase (SDH), responsible for converting succinate into fumarate as part of OXPHOS, is inhibited by the metabolite itaconate [76]. Itaconate is derived from the decarboxylation of cis-aconitate, a substrate of the TCA cycle, and it has been described as an important regulatory metabolite of inflammation, modulating innate immunity to limit tissue damage and metabolism. Apart from inhibiting SDH, and consequently OXPHOS and reactive oxygen species (ROS) generation, it also decreases glycolysis by inhibiting key enzymes, i.e., glyceraldehyde-3-phosphate dehydrogenase (GAPDH) and aldolase a (ALDOA). Moreover, it alkylates Kelch-like ECH-associated protein 1 (KEAP1), inducing the release of nuclear factor NERF2, an antioxidant regulator, and has an anti-inflammatory effect by decreasing the levels of inflammatory cytokines [77]. In humans, itaconate has been extensively described in macrophages and recently in liver tissue [78]. Results suggest a potential modulatory effect mediated by itaconate in NET fish, given that the metabolite was upregulated in challenged fish, along with a significant downregulation of GAPDH (Figure 6). Several proteins implicated in the immune system were regulated by the net-handling challenge (Table S3), including six-transmembrane epithelial antigen of the prostate 4 (STEAP4), a metalloreductase involved in iron and copper homeostasis [79], which was significantly affected by all challenges, i.e., OC, NET, and HYP. Currently, we are unable to corroborate the regulatory effect and importance of itaconate in gilthead seabream metabolism and immunity after stress exposure. However, these are interesting data in light of the evidence that itaconate is such an important regulatory metabolite in humans.

## 4. Materials and Methods

### 4.1. Ethics

The present study was officially approved by the Responsible Body for Animal Welfare (ORBEA) of CCMAR and the Portuguese National Authority for the Animal Health (DGAV), on 26 August 2019. The animal experiments followed the European guidelines on the protection of animals used for scientific purposes (Directive 2010/63/EU) and Portuguese legislation for the use of laboratory animals, under a “Group 1” license (permit number 0420/000/000-n.99–09/11/2009) from the Veterinary Medicine Directorate, the competent Portuguese authority for the protection of animals, Ministry of Agriculture, Rural, Development and Fisheries, following the category C FELASA recommendations.

### 4.2. Fish and Stocking Conditions

*Sparus aurata* adults were randomly distributed in 500 L fiberglass tanks provided with flowthrough seawater from Ria Formosa. Physicochemical parameters varied according to natural fluctuations (natural photoperiod, water temperature at 13.4 ± 2.2 °C, salinity at 34.7 ± 0.8%, and dissolved oxygen level above 5 mg L^−1^). Fish were fed once daily, by hand, with a commercial feed (Standard Orange 6) from “AquaSoja, Sorgal, S.A” (Ovar, Portugal), according to the species’ nutritional requirements. Experimental trials took place at the Ramalhete Research Station of CCMAR (Faro, Portugal), and fish were supplied by the company “Maresa, Mariscos de Estero S.A.” (Huelva, Spain).

### 4.3. Experimental Design

Three separate experimental trials were conducted, where fish were subjected to three challenges: OC—overcrowding; NET—repetitive net handling, coupled with air exposure; and HYP—hypoxia. Two experimental groups were set for each trial: (1) control group (CTRL) and (2) challenged group. Triplicate tanks were used for each group (i.e., a total of six tanks per trial), with an initial rearing density of 10 kg m^−3^ (except for the high-rearing-density group, as further described). In the OC trial, fish with average initial body weights (IBW) of 373.89 ± 11.04 g were subjected to high stocking densities of over 54 days. Experimental groups were established as follows: (1) CTRL weighed 10 kg m^−3^ and (2) OC45 weihed 45 kg m^−3^. In the NET trial, fish (IBW = 376.52 ± 8.96 g) were challenged for 45 days with specific nets designed for the purpose: (1) CTRL referred to undisturbed fish (the net was likewise fit inside the tanks but not lifted) and (2) NET4 referred to fish netted four times a week (fish were lifted and air-exposed for 1 min). The last air-exposure challenge was performed 72 h before sampling. In the HYP trial, fish (IBW = 405.74 ± 35.14 g) experienced low levels of saturated oxygen in the water over 48 h. Experimental groups were established as follows: (1) CTRL had 100% saturated oxygen and (2) HYP15 had 15% saturated oxygen. Measurements of saturated oxygen levels were conducted every 30 min to keep track of potential fluctuations and adjust nitrogen injection if necessary. Zootechnical results were previously published by the authors [32].

### 4.4. Sampling

At the end of each trial, fish were starved for 48 h to clean the digestive tract, according to normal practice in aquaculture production. Three fish were randomly sampled from each tank and immediately anesthetized with a lethal dose of tricaine methanesulfonate (MS-222; Merck KGaA, Darmstadt, Germany). Liver samples were collected, chopped, divided in two Eppendorf tubes (i.e., one for proteomics and one for metabolomics), immediately frozen in liquid nitrogen, and stored at −80 °C until further use.

### 4.5. Sample Preparation

For proteomics analysis, liver protein extracts (n = 6, two fish per tank; tank unit as biological replicate) were obtained from 50 mg of tissue solubilized with 500 μL of extraction buffer (7 M urea, 2 M thiourea, 4% CHAPS, 30 mM Tris pH 8.5), 5 μL of proteases’ inhibitor (PI) cocktail (Merck KGaA), and 2 μL of 250 mM EDTA. Sample homogenization was conducted by using a tissue lyser (VWR, Radnor, PA, USA) with 5 mm metal beads for two cycles of 30 s, at a frequency of 25/s. Homogenates were incubated at 4 °C for 30 min, in constant rotation and centrifuged at 13,000× *g* and 4 °C for 15 min to remove insoluble material. Liver extracts were diluted in the initial buffer, and the protein content was measured by using the BioRad Quick Start Bradford Dye Reagent and BSA Standard Set (Bio-Rad Laboratories, Hercules, CA, USA). Extracts were then depleted of nonprotein contaminants by using the ReadyPrep™ 2D Clean-Up kit (Bio-Rad), following the manufacturer’s instructions. The cleaned protein pellet was further resuspended in 100 mM Tris pH 8.5, 1% sodium deoxycholate, 10 mM TCEP, 40 mM chloroacetamide, and protease inhibitors for 10 min at 95 °C at 1000 rpm (Thermomixer, Eppendorf, Hamburg, Germany). Subsequently, samples were prepared according to the solid-phase-enhanced sample-preparation (SP3) protocol [80]. Enzymatic digestion was conducted with 2 µg Trypsin/LysC overnight at 37 °C at 1000 rpm.

For metabolomics analysis, 10 mg of liver tissue (n = 9, three fish per tank; tank unit as biological replicate) were homogenized on a tissue lyser, with chloroform/methanol/water 1:3:1 (*v/v/v*) at 4 °C and a 5 mm metal bead, for 30 s, at 25/s frequency. Homogenates were incubated at 4 °C for 1 h, in constant rotation, and lastly centrifuged for 3 min at 13,000× *g* and 4 °C. The supernatant was collected and analyzed by LC-MS/MS. In addition, 10 μL from each supernatant sample was pooled as the quality control (QC) sample.

### 4.6. Label-Free Shotgun Proteomics Analysis

#### 4.6.1. NanoLC-MS/MS Analysis

Peptides from enzymatic digestion (500 ng) were analyzed through online nanoLC using an UltiMate TM 3000 system coupled with a Q-Exactive Hybrid Quadrupole-Orbitrap mass spectrometer (Thermo Scientific, Bremen, Germany). Samples were loaded onto a trapping cartridge (Acclaim PepMap C18 100 Å, 5 mm × 300 µm i.d., 160454, Thermo Scientific) in the mobile phase (2% ACN, 0.1% FA at 10 µL min^−1^). After 3 min loading, the trap column was switched inline to a 50 cm by 75 μm inner-diameter EASY-Spray column (ES803, PepMap RSLC, C18, 2 μm, Thermo Scientific) at 250 nL min^−1^. Separation was achieved by mixing A: 0.1% FA and B: 80% ACN, 0.1% FA, with the following gradient: 5 min (2.5% B to 10% B), 120 min (10% B to 30% B), 20 min (30% B to 50% B), 5 min (50% B to 99% B), and 10 min (hold 99% B). Subsequently, the column was equilibrated with 2.5% B for 17 min. Data acquisition was achieved by using Xcalibur 4.0 and Tune 2.9 software (Thermo Scientific).

The mass spectrometer was operated in data-dependent acquisition (DDA), in positive mode, alternating between a full scan (m:s 380–1580) and a subsequent HCD MS/MS of the 10 most-intense peaks from the full scan (normalized collision energy of 27%). The ESI spray voltage was 1.9 kV and capillary temperature was 275 °C. Global settings were: use lock masses best (m:s 445.12003), lock-mass injection full MS, and chromatography peak width (FWHM) of 15 s. Full scan settings: 70k resolution (m:s 200), AGC target 3e6, and maximum injection time 120 ms. DDA settings: minimum AGC target 8e3, intensity threshold 7.3e4. Charge exclusion: unassigned, 1, 8, >8, peptide match preferred, exclude isotopes on, dynamic exclusion 45 s. MS2 settings: microscans 1, resolution 35k (m:s 200), AGC target 2e5, maximum injection time 110 ms, isolation window 2.0 m:s, isolation offset 0.0 m:s, dynamic first mass, and spectrum data–type profile. MS analyses were performed at the Proteomics Scientific Platform of i3S, Porto, Portugal.

#### 4.6.2. Protein Identification

Raw MS data were processed by using SEQUEST^®^ on Proteome Discoverer^TM^ software 2.5.0.400 (Thermo Scientific) and searched against the UniProtKB Eupercaria database (taxon ID 1489922; Release 2020_05; 651,914 sequences). The SEQUEST HT search engine was used for the identification of tryptic peptides. The ion mass tolerance was set at 10 ppm for precursor ions and 0.02 Da for fragmented ions. A maximum of two missed cleavage sites was allowed, with a minimum peptide length of six amino acids and 144 as maximum. Cysteine carbamidomethylation was defined as constant modification. Methionine oxidation, protein N-terminus acetylation, and loss of methionine and Met-loss+Acetyl were defined as variable modifications. Peptide confidence was set to high. The Inferys rescoring node was considered for this analysis. The processing node Percolator was enabled with the following settings: maximum delta Cn 0.05; decoy database search target FDR ≤ 1%, validation based on *q*-value. Protein label-free quantitation was performed with the Minora feature detector node at the processing step. Precursor ions quantification (TOP3) was performed at the processing step with the following parameters: peptides to use unique plus razor, precursor abundance was based on intensity, normalization mode was based on total peptide amount, and the pairwise protein ratio calculation and hypothesis test were based on a *t*-test (background based). Magellan storage files (.msf) were imported into Scaffold v.4.11.1 (Proteome Software Inc.) to validate MS/MS-based peptide and protein identifications. A second search engine, i.e., X! Tandem algorithm (the GPM, thegpm.org; version X! Tandem Alanine 2017.2.1.4), was applied to all MS/MS samples, by using a reverse-concatenated subset of the UniProtKB Eupercaria database (release 2020_05; 651,914 entries), also assuming Trypsin digestion and parent ion tolerance of 10 ppm and a fragment ion mass tolerance of 0.020 Da. Carbamidomethyl of cysteine was specified as a fixed modification and Glu->pyro-Glu of the N-terminus, ammonia-loss of the N-terminus, gln->pyro-Glu of the N-terminus, oxidation of methionine and acetyl of the N-terminus as variable modifications. Peptide identifications were accepted if they could be established at a greater than 92% probability to achieve an FDR less than 0.1% by the peptide prophet algorithm [81] with Scaffold delta-mass correction; protein identifications were accepted at a minimum 99% probability to achieve an FDR less than 1% by the protein prophet algorithm [82], containing at least three identified peptides. Proteins containing similar peptides that could not be differentiated on the basis of MS/MS analysis alone were grouped to satisfy the principles of parsimony. The MS proteomics data were deposited on the ProteomeXchange Consortium [83] via the PRIDE [84] partner repository with the dataset identifier PXD036392 and 10.6019/PXD036392.

### 4.7. Untargeted Metabolomics Analysis

All samples were analyzed on a Thermo Scientific Q-Exactive Orbitrap mass spectrometer, running in positive/negative switching mode, connected to a Dionex UltiMate^TM^ 3000 RSLC system (Thermo Fisher Scientific, Hemel Hempstead, UK) using a ZIC-pHILIC column (150 mm × 4.6 mm, 5 μm column, Merck Sequant, Gillingham, UK). The column was maintained at 30 °C, and samples were eluted with a linear gradient (20 mM ammonium carbonate in water for A and acetonitrile for B) over 46 min at a flow rate of 0.3 mL/min as follows: 0 min 20% A, 30 min 80% A, 31 min 92% A, 36 min 92% A, 37 min 20% A, and 46 min 20% A. The injection volume was 10 μL, and samples were maintained at 5 °C prior to injection. The MS settings were as follows: resolution 70,000, AGC 1e6, m:s range 70–1050, sheath gas 40, auxiliary gas 5, sweep gas 1, probe temperature 150 °C, and capillary temperature 320 °C. For positive mode ionization: source voltage +3.8 kV, S-Lens RF Level 30.00, S-Lens Voltage 25.00 (V), Skimmer Voltage 15.00 (V), Inject Flatopole Offset 8.00 (V), and Bent Flatopole DC 6.00 (V). For negative mode ionization: source voltage was 3.8 kV. The calibration mass range was extended to cover small metabolites by including low-mass calibrants with the standard Thermo calmix masses (below m:s 138): butylamine (C4H11N1) for positive ion electrospray ionization (PIESI) mode (m:s 74.096426) and COF3 for negative ion electrospray ionization (NIESI) mode (m:s 84.9906726). To enhance calibration stability, lock-mass correction was also applied to each analytical run (positive mode: lock mass (m:s) 144.9822; negative mode: lock mass (m:s) 100.9856). To assess the running of the instrument, the pooled samples (QC) were assessed for reproducibility and were run throughout the sample analysis, every fifth sample.

The raw mass spectrometry data were preprocessed using the polyomics-integrated metabolomics pipeline (PiMP) [85]. Briefly, data were converted from Thermo proprietary raw files (.raw) to the open format mzXML and uploaded to PiMP. Within PiMP, unique signals were extracted by using the XCMS centwave algorithm [86] and matched across biological replicates on the basis of a mass-to-charge ratio (m:s) and retention time. These grouped peaks were then filtered on the basis of relative standard deviation and combined into a single file. The combined sets were then filtered on signal to noise score, minimum intensity, and minimum detections. The final peak set was then gap filled and displayed in the PiMP user interface. Filters were applied using MZMatch [87]. Metabolite identifications were validated by comparing the chromatographic retention times and the m:s values against a set of in-house standards. The metabolites’ level of identification was classified according to the Metabolomics Standards Initiative (MSI) (Table S4) [88]. The MS metabolomics data were deposited on MetaboLights [89] with the dataset identifier MTBLS5940.

### 4.8. Univariate and Multivariate Statistical Analyses

Protein abundance was estimated on the basis of TOP3 precursor intensity, and normalized values (against the sum of all ion intensities in each sample replicate) were log10 transformed prior to statistical analysis [63]. The residuals’ normality and homoscedasticity were confirmed by using the SPSS^TM^ Statistics software v.25.0 (IBM^TM^, Armonk, NY, USA). Univariate statistical analyses of proteomics data were performed in the Perseus software [90], including only proteins quantified in at least four out of six replicates per experimental group. Differences in protein abundance between control group and experimental group, within each trial, were analyzed by using a Student’s *t*-test (*p* < 0.05) with FDR controlled at 0.05. Statistical analyses of the metabolomics data were performed on MetaboAnalyst 5.0 (https://www.metaboanalyst.ca/home.xhtml) [91], using only the metabolites detected in at least six out of nine replicates per experimental group. Prior to univariate hypothesis testing, peak intensities were log10 transformed and Pareto scaled, and the missing values were imputed on the basis of one-fifth of the minimum positive value of each variable. Differential analysis was achieved by a Student’s *t*-test (*p* < 0.05) with FDR controlled at 0.05.

A multivariate statistical analysis was performed by using R v.4.2.0 [92] for MacOSX. Group separation and replicates’ variability were analyzed, for each data modality, through PCA, using the prcomp R function in the autoscaled matrices. Missing values were imputed on the basis of the feature median. Biplots were generated by using the R package factoextra [93]. Data integration analysis for biomarker discovery using latent components (DIABLO), within the R package *mixOmics* [94], was used to perform the integrative analysis of proteomics and metabolomics datasets (identified proteins and metabolites) and depict the 10 most-correlated and -discriminatory features between both data modalities. The parameters of the final DIABLO model were tuned based on three-fold cross validation repeated five times and using the centroids distance as distance metric.

### 4.9. Functional Analyses

Joint pathway analysis (JPA) of DAPs and DAMs was performed on the MetaboAnalyst 5.0, using the Entrez IDs for proteins and compound KEGG IDs for metabolites as queries and *Danio rerio* as reference. Overrepresentation analysis (ORA) was achieved with two-sided hypergeometric test, followed by Benjamini-Hochberg method for *p*-value correction and using the combined *p*-values at the pathway level as integration method. Enriched terms and the corresponding features were visualized on a cnetplot using the R package *enrichplot* [95].

The search tool for the interactions of chemicals (STITCH) [96] was used to generate, for each trial, a metabolic reaction network (protein-metabolite) of DAPs and DAMs. Protein identifiers from the STRING database [97] and compound names were used as queries. Networks were visualized and topologically analyzed with the Cytoscape software v.3.8.1 [98], using the plugin MCODE [99] to extract significant clusters from the main network according to the default parameters.

Enriched REACTOME terms within the features selected by the DIABLO model were depicted by using the analysis tool from the knowledgebase REACTOME [100], with the identifiers projected to human orthologues as queries and visualized as Voronoi plots.

All figures were created by using the open-source graphics editor Inkscape (http://www.inkscape.org/).

## 5. Conclusions

The fish stress response has been extensively studied at different levels, e.g., behavioral changes, physiological indicators, metabolic adaptations, immune responses, oxidative stress, the genomic and nongenomic effects of glucocorticoids, etc. However, the regulatory mechanisms of cellular stress are less explored, and studies addressing the cellular stress response at different organizational levels are scarce. Stress-related signaling pathways are evolutionarily conserved, playing an important role in maintaining homeostasis and ensuring cell survival.

In this multiomics study, we demonstrated that the gilthead seabream responded to cellular stress through an intricate network of regulatory components, modulating different signaling pathways to different extents, according to the stress nature. Further, mTORC1 signaling appears to be the crosstalk between ER stress and metabolic reprogramming, stimulating/inhibiting a myriad of biosynthetic and catabolic processes, otherwise maintained at basal homeostatic states, depending on the cell resources’ availability. Net-handled fish appear to have activated the UPR to deal with an ER flooded with unfolded/misfolded proteins, shutting down most metabolic pathways to shift the energy toward the translation of stress-related mRNAs. Contrarily, hypoxia-exposed fish, because of the low availability of cellular energy, appear to have downregulated protein synthesis in an mTORC1-dependent manner. Notably, endocytosis was an upregulated shared pathway between NET and HYP fish, suggesting an important role in stress homeostasis. Fish exposed to overcrowding showed lower alterations at the proteome level and no significant alterations at the metabolome level, suggesting that gilthead seabream might be more resistant to high rearing densities than physical stresses or low oxygen availability is. Additionally, the integrated analysis depicted the most dysregulated pathways, along with the interconnections between the proteins and the metabolites that were responding to the different challenges. This study shows that both omics validated and complemented each other, presenting a clear advantage over more-conventional single-omics analyses and paving the way to more-holistic approaches in fish stress research. These findings provide interesting new avenues of investigation for the study of unbiased fish welfare biomarkers, which will not only impact the aquaculture industry resilience by offering better decision-making and stress prevention strategies but also offer higher food safety from aquaculture products.

## Data Availability

Liver proteomics and metabolomics mass spectrometry data have been made fully accessible via ProteomeXchange, with the identifier PXD036392, and via MetaboLights, with the identifier MTBLS5940.

## Supplementary Materials

The supporting information can be downloaded at https://www.mdpi.com/article/10.3390/ijms232315395/s1.
