# Supplementary material for: Gilthead Seabream Liver Integrative Proteomics and Metabolomics Analysis Reveals Regulation by Different Prosurvival Pathways in the Metabolic Adaptation to Stress
Source: Int J Mol Sci. 2022 Dec 6;23(23):15395. doi: 10.3390/ijms232315395 (PMC9741202; doi:10.3390/ijms232315395)
Supplement: Supplementary file 1 [file ijms-23-15395-s001.zip › Supplemental figures.pdf]

Supplemental figures

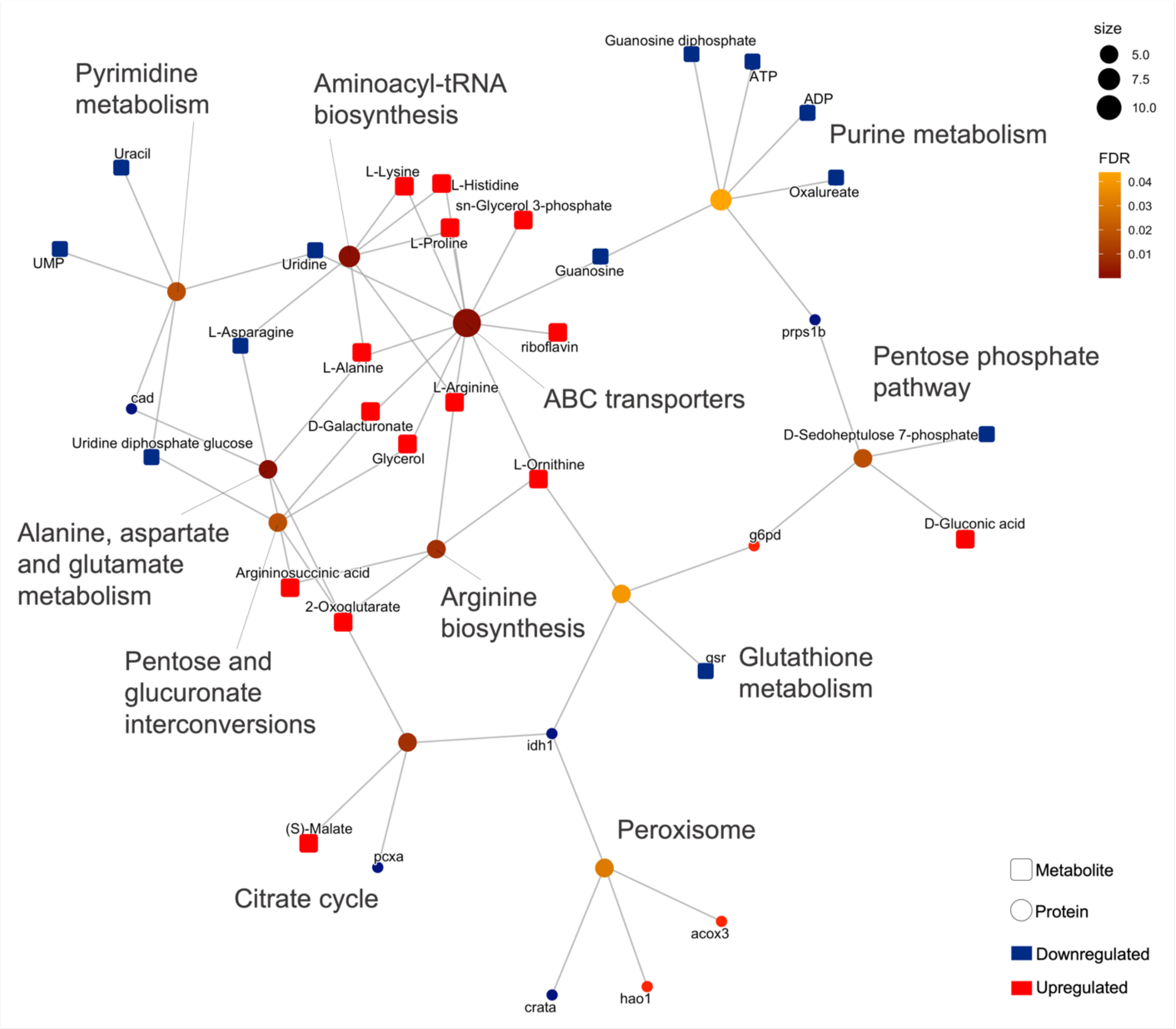

Figure S1. Gene-concept network of enriched KEGG terms (FDR < 0.05) within the differential abundant proteins (DAPs) and metabolites (DAMs) identified in the liver of gilthead seabream submitted to a hypoxia challenge.

Central nodes represent the enriched term, with color and size representing FDR and the number of associated biomolecules, respectively. The concept nodes represent biological concepts, where shape corresponds to the omics modality and color to the regulation of that biomolecule, determined by Student's t-test with FDR controlled at 0.05.

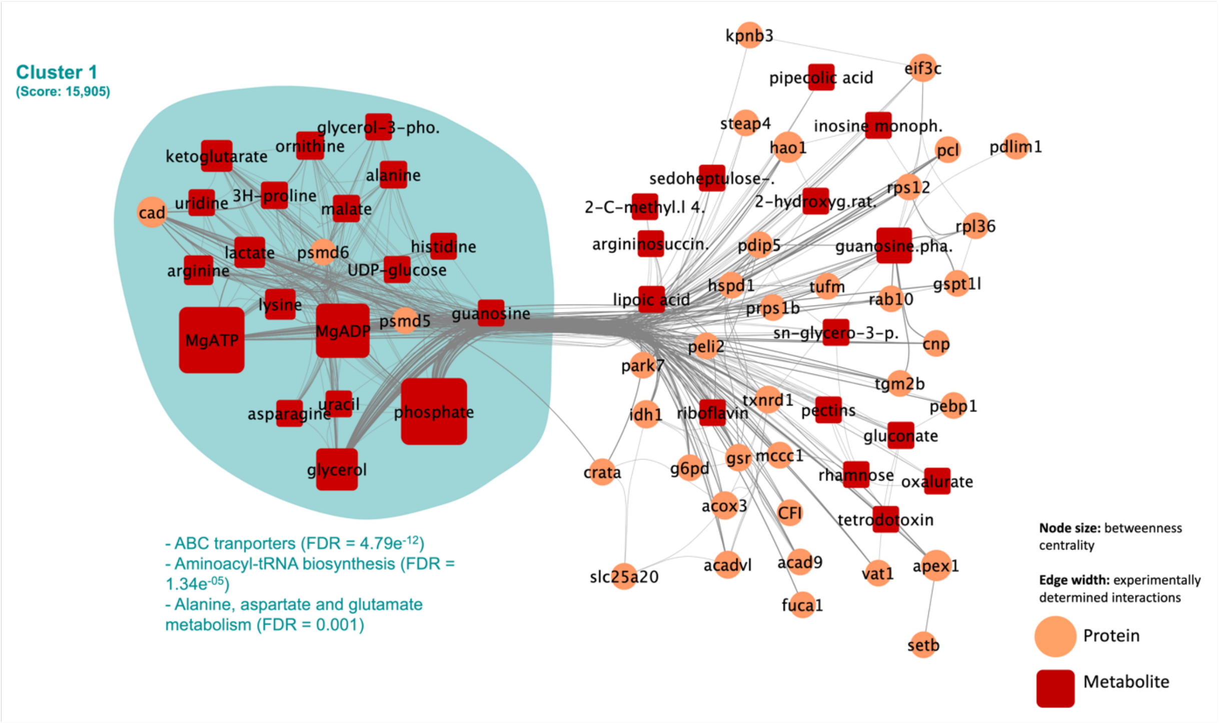

Figure S2. Metabolic reaction network generated with the differential abundant proteins and metabolites identified in the liver of gilthead seabream submitted to a hypoxia challenge. Node shape and color represent the type of biomolecule, according to the legend. Edges represent functional linkages between them. The highlighted cluster, depicted with MCODE plugin within Cytoscape software, represent the most interconnected region, with the corresponding overrepresented KEGG terms (FDR < 0.05).

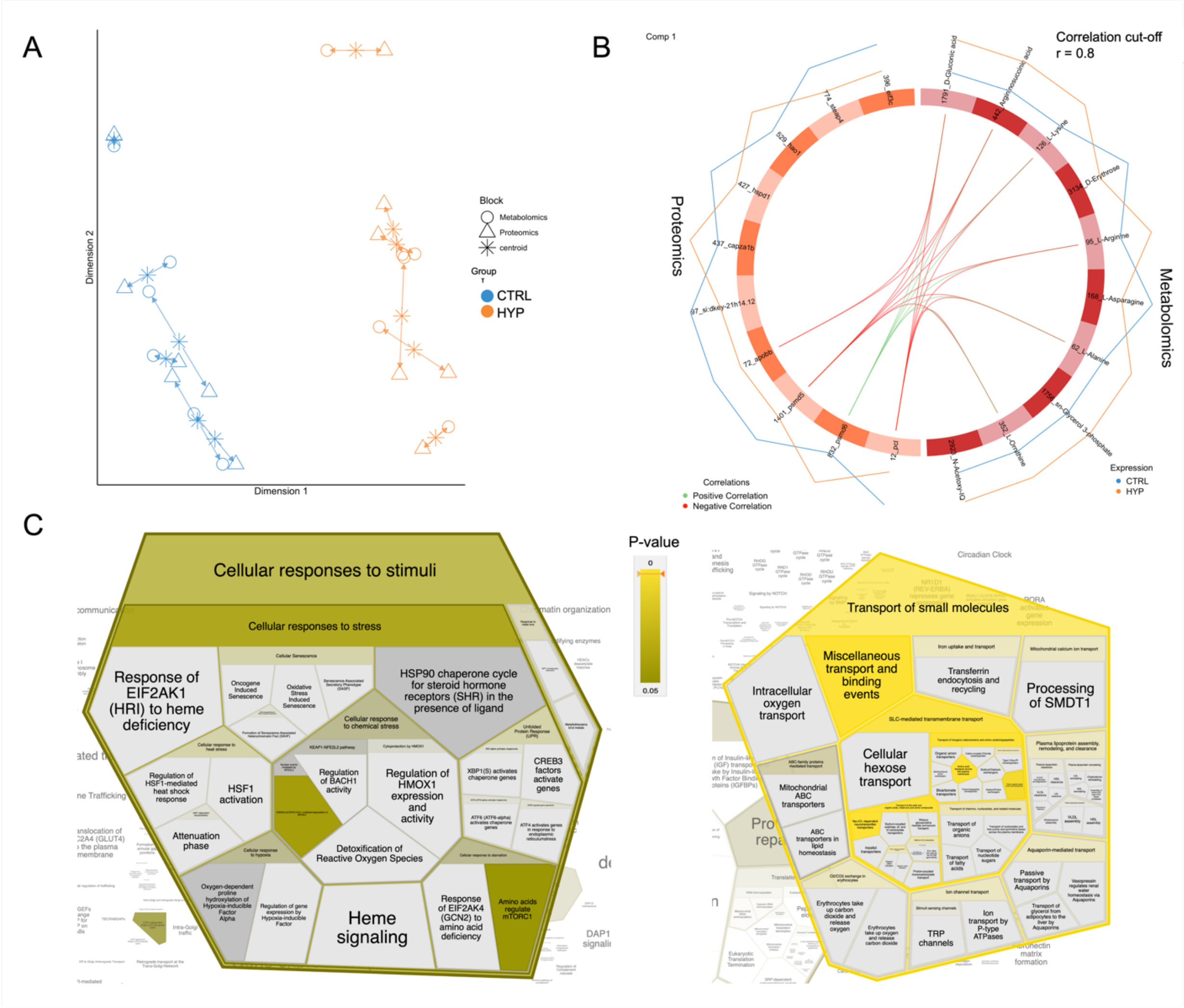

Figure S3. Integrated proteomics and metabolomics analysis, performed with DIABLO, of the liver of gilthead seabream submitted to a hypoxia challenge.

(A) Arrow plot of the separation between groups achieved with the first two components of the DIABLO model. Different shapes represent different data modalities. (B) Circos plots representing the Pearson correlation (correlation cutoff = 0.8) between the ten most discriminatory proteins and metabolites selected by the first component of the DIABLO model. (C) Voronoi plots obtained with REACTOME analysis tool, representing two of the most overrepresented high category terms (FDR < 0.05), among up-regulated features.
